# Supplementary material for: Diagnostic accuracy of self-reported food consumption and shaking chills in predicting bacteremia in outpatients: A prospective, multicenter observational study
Source: PLoS One. 2025 Jul 8;20(7):e0327789. doi: 10.1371/journal.pone.0327789 (PMC12237268; doi:10.1371/journal.pone.0327789)
Supplement: Attachment — Submitted filename: Response_to_Reviewers.docx [file pone.0327789.s002.docx]

February 28, 2025

Professor Adriana Calderaro

Academic Editor

*PLOS ONE*

Dear Professor Calderaro:

Thank you very much for your review of our manuscript entitled **“Diagnostic accuracy of self-reported food consumption and shaking chills in predicting bacteremia in outpatients: a prospective, multicenter observational study”**. First, we apologize for the insufficient points in the journal requirements. We edited our manuscript, including those points. Moreover, we appreciate the comments provided and, in response to these comments, have revised the manuscript PONE-D-24-58497 as much as we could.

The following is a point-by-point reply to the Journal Requirements and the reviewer’s comments as follows:

We hope that we have adequately addressed the concerns of the editor and reviewers. We believe that the manuscript is significantly improved and hope that you share our enthusiasm. We look forward to a publication of our manuscript in *PLOS ONE*.

Sincerely yours,

Takayuki Komatsu

Department of Emergency and Critical Care Medicine,

Juntendo University Nerima Hospital, Tokyo, Japan,

Postal address: 2-1-1 Hongo, Bunkyo-ku,

Tokyo 113-8421, Japan

Tel.: +81-3-5802-1937;

Fax: +81-3-5802-1937;

Email: [tkomatu@juntendo.ac.jp](mailto:tkomatu@juntendo.ac.jp);

ORCID: 0000-0002-8730-2081

**Journal Requirements:**
**Comment 1)** Please ensure that your manuscript meets PLOS ONE’s style requirements, including those for file naming.

**Reply 1:** Thank you very much for your comment. We have rechecked the PLOS ONE style templates and revised the manuscript accordingly.

**Comment 2:** Please state what role the funders took in the study. If the funders had no role, please state: “The funders had no role in study design, data collection and analysis, decision to publish, or preparation of the manuscript.” If this statement is not correct you must amend it as needed. Please include this amended Role of Funder statement in your cover letter; we will change the online submission form on your behalf.

**Reply 2:** Thank you very much for your suggestion. We have added the following sentences at the end of the revised cover letter: “This study was funded by JSPS KAKENHI Grant-in-Aid for Early-Career Scientists (Grant number JP19K16990). The funders had no role in study design, data collection and analysis, decision to publish, or preparation of the manuscript.”

**Comment 3)** In this instance it seems there may be acceptable restrictions in place that prevent the public sharing of your minimal data. However, in line with our goal of ensuring long-term data availability to all interested researchers, PLOS’ Data Policy states that authors cannot be the sole named individuals responsible for ensuring data access ([http://journals.plos.org/plosone/s/data-availability#loc-acceptable-data-sharing-methods](http://track.editorialmanager.com/CL0/http:%2F%2Fjournals.plos.org%2Fplosone%2Fs%2Fdata-availability%23loc-acceptable-data-sharing-methods/1/010f01946a8180f1-c08b915a-a758-48f7-befc-abefd8475077-000000/ZUJF6ORVlKWvRZdycS07T0Quhn7IoS4nDtr8vGXckHU=194)). Data requests to a non-author institutional point of contact, such as a data access or ethics committee, helps guarantee long term stability and availability of data. Providing interested researchers with a durable point of contact ensures data will be accessible even if an author changes email addresses, institutions, or becomes unavailable to answer requests.

Before we proceed with your manuscript, please also provide non-author contact information (phone/email/hyperlink) for a data access committee, ethics committee, or other institutional body to which data requests may be sent. If no institutional body is available to respond to requests for your minimal data, please consider if there any institutional representatives who did not collaborate in the study, and are not listed as authors on the manuscript, who would be able to hold the data and respond to external requests for data access? If so, please provide their contact information (i.e., email address). Please also provide details on how you will ensure persistent or long-term data storage and availability.

**Reply 3:** Thank you for pointing out these important requirements. We have revised our Data Availability Statement to address these concerns. Masashi Nagao, at the Clinical Research & Trial Center, Juntendo University, has been designated as the institutional data manager (nagao@juntendo.ac.jp).

**Reviewer(s)’ Comments to Author:**

**Reviewer: #1**

**Comments to the Author:**

I would like to commend the authors for conducting this valuable study and for the opportunity to review it. Developing simple and practical algorithms to predict bacteremia, especially patient-friendly ones, is crucial for reducing sepsis-related mortality and morbidity. This article evaluates the utility of “self-reported” food consumption and shaking chills immediately before taking blood cultures (BC) in predicting the presence or absence of bacteremia in outpatients, primarily those arriving by ambulance. The findings suggest that these self-reported factors are useful predictors of bacteremia in this population. The study’s objectives and clinical implications are clear, and its results hold potential relevance for both patients and practitioners. However, revisions are necessary to enhance the scientific rigor and ensure readers can interpret the findings appropriately.

**Reply:** Thank you for your insightful comments and for recognizing the value of our study. We appreciate your constructive feedback and have revised the manuscript accordingly to enhance its scientific rigor.

**Major comments**

**Comment 1)** Inclusion criteria

The authors assessed the diagnostic accuracy of food consumption and shaking chills separately. It would be helpful to clarify why patients who reported only food consumption or shaking chills were excluded from the analysis. Please provide the rationale for this decision and discuss how it might impact the generalizability of the findings.

**Reply 1:** Thank you for your insightful comment. We apologize for the insufficient explanation. In our previous study (Komatsu T, et al., J Hosp Med 2017), we observed that some patients with bacteremia maintained normal food consumption, regardless of whether they experienced shaking chills. Based on this finding, we concluded that both self-reported food consumption and shaking chills were necessary to fully assess their combined predictive value in identifying bacteremia.
We have now clarified this rationale in the subsection “Exclusion Criteria” (Page 7, Lines 110–114) of the “Materials and Methods” section. Additionally, we recognize that this exclusion criterion may affect the generalizability of our findings. To address this, we have incorporated a discussion of this limitation by combining the fourth and final limitations into a single revised fourth limitation in the “Discussion” section (Page 29, Line 355–Page 30, Line 363).

**Comment 2)** Potential confounding

If available, please provide data on the time elapsed between symptom onset and BC collection. Since “food consumption immediately before BC collection” could be influenced by this time interval, understanding its impact on the study results is critical.

**Reply 2:** Thank you for your insightful comment. As you correctly pointed out, understanding the time interval between symptom onset, the last meal, and blood culture (BC) collection is critical for interpreting the diagnostic accuracy of self-reported food consumption. However, we did not collect this information in the questionnaire, as accurately determining the timing of symptom onset was challenging for many patients.
To address this limitation, we have now clarified this point in the “Materials and Methods” section by adding “(i.e., last meal)” to the “Food Consumption” subsection (Page 9, Line 153).

**Comment 3)** Impact of predictors on BC collection decisions

Physicians’ awareness of the predictors (e.g., food consumption and shaking chills) could have influenced their decision to collect BC. If this information was accessible to physicians before BC collection, it may introduce bias. To address this, the authors might consider discussing whether the prevalence of bacteremia observed in this study (12.7%) aligns with rates reported in similar settings. This comparison could help assess the potential bias in the study results.

**Reply 3:** Thank you for your thoughtful comment. Since this study was conducted in a clinical setting, physicians made decisions regarding blood culture (BC) collection based on their usual clinical reasoning, which included patient history, physical examination, and laboratory findings. As a result, physicians were naturally aware of the predictors (food consumption and shaking chills) before deciding whether to collect BCs, which could have introduced a potential selection bias.
To assess the impact of this possible bias, we compared the prevalence of bacteremia in our study (12.7%) with rates observed in similar studies. Notably, our previous study, which used the same decision-making process for BC collection, reported a bacteremia prevalence of 12.0% (221/1,847). Additionally, a study conducted in an emergency department (Rothe K, et al., BMC Infect Dis 2019) found a prevalence of 14.3%. These comparable rates suggest that physician decision-making in our study was consistent with established clinical practice and likely did not introduce significant bias.
We have incorporated these points into the “Collecting Blood Cultures” subsection in the Methods section (Page 7, Lines 118–120), the “Patient Characteristics” subsection in the Results section (Page 12, Line 204), and the Discussion section (Page 29, Lines 345–350), where this issue is addressed as a second limitation.

**Comment 4)** Multiple logistic regression models

To assess the diagnostic performance of food consumption and shaking chills, it would be appropriate to use multiple logistic regression models that adjust for potential confounding variables. Instead of employing forward or backward stepwise selection, variable inclusion should be guided by a conceptual model outlining plausible confounders. Additionally, given that over 10% of patients were excluded from the analysis due to missing data, the authors should address the potential impact of these exclusions. Performing sensitivity analyses using imputation methods to handle missing data could strengthen the robustness of the findings.

**Reply 4:** Thank you for your insightful comment. We acknowledge the importance of using a multiple logistic regression model that adjusts for potential confounders based on a conceptual framework. While we initially considered using imputation methods to address missing data, accurately calculating the Systemic Inflammatory Response Syndrome (SIRS) and quick Sequential Organ Failure Assessment (qSOFA) scores proved challenging due to inaccurate data on components that make up the SIRS criteria (body temperature, heart rate, respiratory rate, and white blood cell count) and the qSOFA score (Glasgow Coma Scale, systolic blood pressure, and respiratory rate). Since these variables play a critical role in predicting bacteremia, any imputation might have introduced substantial inaccuracies in classifying patients as having SIRS or qSOFA ≥2. As a result, we opted for a complete-case analysis, acknowledging the trade-off involved.
To clarify our approach, we have added explanations regarding the components of SIRS and qSOFA in the “Other Predictive Variables” subsection (Page 10, Lines 174–177) and the “Statistical Analysis” subsection (Page 11, Lines 189–194) of the Materials and Methods section.
Additionally, we acknowledge that 83 patients were excluded from the multiple logistic regression analysis due to missing vital sign data. To assess the potential impact of these exclusions, we examined the characteristics of these patients. Notably, except for one case, none were transported by ambulance, suggesting that they were likely not in critically ill condition. Therefore, while their exclusion may have led to some degree of selection bias, the overall effect on the association between SIRS/qSOFA and bacteremia is expected to be minimal. We have now included this discussion in the Discussion section (Page 28, Lines 322–329).

**Minor comments**

**Comment 1)** Guidelines

Since the article focuses on diagnostic performance, adherence to the STARD guidelines would be more appropriate than STROBE. Please consider revising the reporting accordingly.

**Reply 1:** Thank you for pointing this out. We acknowledge that the STARD guidelines are more appropriate for reporting diagnostic performance studies than the STROBE guidelines. In response to your suggestion, we have revised the manuscript to align with STARD recommendations where applicable. Specifically, we have updated the “Ethics Approval” subsection (Page 7, Lines 103–104) in the Materials and Methods section to reflect this change. Additionally, we have reviewed the manuscript to ensure adherence to key elements of the STARD checklist, including clear reporting of study design, patient selection, index and reference tests, and diagnostic accuracy measures.

**Comment 2)** Interrater reliability for the assessment of contamination

The assessment of blood culture contamination is an important aspect of the study. Please provide data on the interrater reliability of this assessment to ensure the consistency and validity of these findings.

**Reply 2:** Thank you for your comment. We apologize for the insufficient information. In this study, contamination was assessed based on a consensus decision by the infection control team as part of routine medical practice. While formal interrater reliability metrics were not calculated, the determination of contamination followed predefined institutional criteria to ensure consistency. These criteria included the type of organism isolated, clinical correlation with the patient’s presentation, and laboratory findings.
Since there were no disagreements among the infection control team members, calculating interrater reliability was not necessary. However, to clarify this process, we have added more details about the definition and assessment of contamination in the “Definition of True Bacteremia and Contamination” subsection (Page 8, Lines 131–141) of the Materials and Methods section.

**Reviewer: #2**

**Comments to the Author:**

Thank you for the opportunity to review this study. The authors have investigated the relationship between dietary intake and the positivity rate of blood cultures. I would like to point out several concerns to improve the quality of this study.

**Reply:** Thank you very much for your comments and for taking the time to review our manuscript. We appreciate your concerns regarding the relationship between dietary intake and the positivity rate of blood cultures, and we have carefully considered each of the points you raised. We believe that these revisions have improved the quality of our study and addressed the issues you identified. Thank you again for your thoughtful feedback, which has helped us strengthen our manuscript.

**Mager comments:**

**Comment 1)** Abstract: Even if a blood culture is a false positive, it is not difficult to determine contamination, and false negatives have a greater impact. Therefore, efforts should be made to reduce false negatives. Poor food consumption had a negative likelihood ratio of 0.66 (95% CI: 0.23-1.94). As the authors wrote it in the limitation, the food consumption was only useful for blood cultures collected between 10 pm and 8 am. A negative predictive value between 8 am and 10 pm was 0.32 (0.12-0.89). This figure was not so low, and it is insufficient to exclude bacteremia.

**Reply 1:** Thank you very much for your insightful comments. We fully agree that the utility of self-reported normal food consumption as a rule-out criterion during daytime hours (8 am – 10 pm) is limited, as indicated by the negative predictive value (0.32, 95% CI: 0.12–0.89).

In response to this, we have revised both the Abstract (Page 4, Lines 46–47) and the Conclusions (Page 31, Lines 376–378) to better reflect this limitation

**Comment 2)** Study design: Please list the names of the three hospitals that participated in the study. Are they all university hospitals? The fact that only 715 blood cultures were ordered in two years is significantly lower compared to community hospitals. Are there few patients who visit without an appointment due to fever?

**Reply 2:** Thank you for your valuable suggestion. In response to your comment, we have added the names and bed capacities of the three participating hospitals in the “Study Design and Patients” subsection (Page 6, Lines 88–91) of the Materials and Methods section.

We regret the omission of an important enrollment criterion. We have now clarified that only outpatients aged >16 years who underwent blood cultures (BC) and completed a medical questionnaire were included in the study (Page 6, Lines 91–92).

Regarding the lower-than-expected number of blood cultures ordered (715 over two years), several factors contributed to this:

1. The two smaller participating hospitals (94 and 99 beds, respectively) are community hospitals, which generally receive fewer walk-in fever patients compared to larger emergency centers.
2. Although the number of febrile patients visiting our hospitals increased during the COVID-19 pandemic, some hospitals originally scheduled to participate were forced to withdraw due to overwhelming clinical demands.
3. Strict infection control protocols during the pandemic limited the administration of medical questionnaires, thereby reducing the number of eligible study participants.

These factors contributed to a smaller sample size than initially anticipated despite exceeding the calculated minimum sample size for the study.

**Comment 3)** Collecting blood cultures: How many sets of blood cultures were collected and from which sites? Is there only one case of contamination with S. capitis? If so, the contamination rate seems very low.

**Reply 3:** Thank you for your comment. In response, we have clarified that at least two sets of blood cultures were collected and have added details regarding the collection sites in the “Collecting Blood Cultures” subsection (Page 7, Line 120–Page 8, Line 124) of the Materials and Methods section.

Regarding the contamination rate, we would like to clarify that the case of *S. capitis* in Table 2 was not a contamination case but rather a pathogen identified in true bacteremia. As shown in Figure 1, the overall contamination rate in our study was 3.9% (21/534 cases). This contamination rate is comparable to previously reported rates from a review article, which was cited in this study (Ref. 19: Hall KK, Lyman JA. Clin Microbiol Rev. 2006;19: 788-802). To improve clarity, we have now included the contamination rate and the number of cases in the “Patient Characteristics” subsection (Page 12, Line 204) of the Results section.

**Comment 4)** Table 6: Why were prior antibiotic use, viral infections, and non-infectious diseases not included as explanatory variables in the multivariate analysis? These clearly affect the positivity rate of blood cultures and should be adjusted for as confounding factors.

**Reply 4:** Thank you for your insightful comment. We apologize for the insufficient explanation. Regarding viral infections and non-infectious diseases, these conditions were only observed in the non-bacteremia group. Since a definitive diagnosis was not always available at the time of blood culture collection, these factors were not considered direct confounders in predicting bacteremia.

As for prior antibiotic use, we did not include it in the multivariate analysis for ruling in bacteremia because it primarily influences the non-bacteremia group, rather than acting as a confounder that affects both groups equally. To address this, we have now clarified the components selected in the multivariate analysis in the Statistical Analysis subsection (Page 11, Lines 189–194) of the Materials and Methods section.

Additionally, we have discussed the positivity rates in response to Reviewer #1, Comment 3, and have added this as a second limitation in the Discussion section (Page 29, Lines 345–350).

**Comment 5)** Discussion: If dietary intake is low, the negative predictive value of blood cultures is high at 91%, but if dietary intake is not low, blood cultures are less likely to be positive. To demonstrate this, it would be easier to understand if the authors show that the positive predictive value of blood cultures is low when dietary intake is adequate.

**Reply 5:** Thank you for your comment. We apologize for any difficulty in understanding our findings. The positive predictive value (PPV) of normal food consumption for predicting bacteremia was 8.8%. However, the sensitivity, specificity, negative predictive value (NPV), positive likelihood ratio (+LR), and negative likelihood ratio (-LR) were 13.2%, 80.0%, 86.3%, 0.66, and 1.08, respectively. These results were thought to complicate the understanding, as none of the diagnostic accuracy measures, including PPV, provided impressive values.

To highlight the usefulness of normal food consumption in ruling out bacteremia, we have revised the Abstract (Page 3, Lines 40–41), the Food Consumption subsection in the Results section (Page 20, Lines 250–253), and the Discussion section (Page 27, Lines 302–303) to emphasize this distinction and ensure that the diagnostic accuracy measures are clearly reported. These changes aim to make the findings more comprehensible and accurately convey the limited role of normal food consumption in predicting bacteremia.

**Comment 6)** Discussion: The importance of chills is the same for both outpatient and inpatient settings. In a university hospital patient group, there are fewer elderly patients and dementia patients, making it difficult to ascertain dietary intake and the presence of chills. Therefore, these research findings may not be applicable to community hospitals. It would be better to add this as a limitation.

**Reply 6:** Thank you for your valuable comment. In response, we have clarified the hospital characteristics in our reply to Comment 2. While Juntendo University Nerima Hospital functions as a university hospital, it also serves as a regional core hospital, receiving a significant number of elderly patients with acute illnesses.

We recognize that assessing dietary intake and the presence of chills in elderly patients, especially those with dementia, is challenging. To address this, our study included older adult patients, and when necessary, medical questionnaires were completed by their representatives. However, we acknowledge that some patients, particularly those with dementia or altered consciousness who did not have a representative, were discouraged from adapting our results. This limitation could affect the generalizability of our findings to hospital settings with a higher proportion of such patients. Since this point is partially related to Reviewer #1’s Comment 1, we have integrated this as the fourth limitation in the Discussion section (Page 30, Lines 355–364).

**Comment 7)** Discussion: Even if dietary intake is adequate, nearly 10% (9/102) still had positive blood cultures. Therefore, even with adequate dietary intake, blood cultures should not be omitted. I am concerned that if you decide not to collect blood cultures from patients who can eat, it may delay the diagnosis of infections that can only be diagnosed through blood cultures, such as infective endocarditis, thereby increasing the risk of poor outcomes for such patients.

**Reply 7:** Thank you for your insightful comment. We completely agree with your concern. We do not intend for physicians to restrict blood culture collection based solely on dietary intake. As you correctly pointed out, even among patients with adequate dietary intake, nearly 10% (9/102) had bacteremia, indicating that blood cultures should not be omitted when clinical suspicion is present. In cases where infective endocarditis, abscesses, or other infections with a direct risk of bacteremia are suspected, especially those with sudden onset or structural risk factors, blood cultures remain essential for accurate diagnosis and timely intervention. We have added this point as the final limitation (Page 30, Lines 364–373).

**Minor comments**

**Comment 1)** Chills: How was “uncertain” classified for chills?

**Reply 1:** Thank you for pointing out the potential confusion regarding the classification of “uncertain” for chills. We apologize for any ambiguity and appreciate your feedback. To clarify, we included the option “uncertain” in our questionnaire to account for cases where patients or their representatives were unable to recall or determine the severity of chills. We have added further clarification in the “Chills” subsection (Page 10, Lines 165–166) of the “Methods” section to address this point.

**Comment 2)** Pathogen and diagnosis: Enterobacteriaceae is now referred to as Enterobacterales.

**Reply 2:** Thank you for highlighting this. In response to Reviewer 2’s comments, we have edited the manuscript in the “Pathogen and Diagnosis” subsection (Page 16, Line 231) of the “Results” section.

**Comment 3)** Pathogen and diagnosis: Which site does the abscess refer to?

**Reply 3:** Thank you for your comments. To clarify, the sites of abscess in our study referred to a variety of locations, including subcutaneous, intra-abdominal, and other tissues. In the bacteremia group, the specific sites of abscess were as follows: two cases of subcutaneous abscesses at the buttock, and one case each of liver, prostate, iliopsoas, and intramuscular abscess at the thigh. In contrast, the non-bacteremia group had a more diverse range of abscess sites, including four cases of subcutaneous abscesses, three cases of intraperitoneal abscesses, two cases of liver abscesses, and one case each of kidney, iliopsoas, intrauterine, intragingival, peritonsillar, perianal, and urachal remnants. We have added a table legend to the “Pathogen and Diagnosis” subsection (Page 19, Lines 240–244) in the “Results” section to provide a clear summary of these findings.

**Comment 4)** Table 2: Only a part of it is labeled as beta-strept. The “beta” is unnecessary.

**Reply 4:** Thank you for pointing out the error in Table 2. In response to your comment, we have revised the table (Page 17) and removed the unnecessary “beta” label. The table now simply refers to “strept.”
