# Supplementary material for: Diagnostic accuracy of self-reported food consumption and shaking chills in predicting bacteremia in outpatients: A prospective, multicenter observational study
Source: PLoS One. 2025 Jul 8;20(7):e0327789. doi: 10.1371/journal.pone.0327789 (PMC12237268; doi:10.1371/journal.pone.0327789)
Supplement: Attachment — Submitted filename: Response to Reviewers.docx [file pone.0327789.s003.docx]

**Reviewer(s)’ Comments to Author:**

**Reviewer: #1**

**Comments to the Author:**

I thank the authors for their thoughtful revision. Most of my previous comments are now addressed; however, I would like the authors to reconsider the rest.

**Reply:** We are very grateful for your constructive feedback. In response to your comments, we have revised the manuscript to enhance its scientific rigor

**Major comments**

**Comment 1)** Inclusion criteria

I am unsure whether the authors’ reason for including only patients who reported both+ food consumption and shaking chills is right. The authors said that “we concluded that

both self-reported food consumption and shaking chills were necessary to fully assess their combined predictive value in identifying bacteremia,” however, I couldn’t find any combined (food consumption and shaking chills) predictive value in identifying bacteremia.

**Reply 1:** Thank you for pointing that out. We sincerely regret the confusion. The sentence, “we concluded that both self-reported food consumption and shaking chills were necessary to fully assess their combined predictive value in identifying bacteremia,” refers to our previous study, not the current one. However, we mistakenly used the term “self-reported” instead of “nurse-assessed.” During the proofreading process, we failed to notice that the content had changed to something different from our original intent. We sincerely apologize for not thoroughly reviewing the document. We are very grateful that you pointed this out.

In the present study, we did not intend to assess the *combined* diagnostic accuracy of these two signs. However, we did believe it was necessary to gather detailed information on patients with bacteremia who had maintained normal food consumption, regardless of whether they exhibited shaking chills. This is because, in a previous study, some bacteremia patients with normal food consumption also had shaking chills, as described in the “Exclusion Criteria” section of the Materials and Methods. Therefore, we required both the food consumption data and the grade of chills.

**Comment 2)** Multiple logistic regression models

The authors did not fully respond to my previous comments about why they did not include variables based on any conceptual model instead of employing forward or backward stepwise selection.

**Reply 2:** Thank you for emphasizing the importance of an a priori conceptual framework for covariate selection. In response to your recommendation, we have re-conducted the multiple logistic regression analysis by including the following variables, which were forced into the model based on our conceptual model of bacteremia risk: sex, CRP >10 mg/dL, shaking chills, and SIRS, all of which have been consistently identified in prior studies [1, 12]. Additionally, age ≥75 years, food consumption, qSOFA score ≥2, method of arrival, and timing of blood culture collection were also analyzed as other covariates.

The revised analysis (Table 6, p.27) yields the following ORs (95% CIs): shaking chills 3.20 (1.53–6.67), age ≥75 years 2.89 (1.41–5.90), SIRS 2.75 (1.28–5.54), CRP >10 mg/dL 2.74 (1.50–4.98), and transportation by ambulance 2.46 (1.17–5.18). These estimates differ only marginally and do not materially affect the interpretation; therefore, no changes were required in the Discussion section.

We have revised the “Statistical Analysis” subsection (p. 12, lines 193–197) of the Materials and Methods section to describe the conceptual model approach and have updated the “Ruling in Bacteremia” subsection (p. 25, lines 287–289; p. 26, lines 290–300) of the Results section.

**Reviewer: #2**

**Comments to the Author:**

Thank you for revising the manuscript. In Table 2, ‘Streptococcus bovis’ is listed, but this is the former name, and the current name is ‘Streptococcus gallolyticus.’ Other than this, there are no particular points that need correction.

**Reply:** Thank you for drawing our attention to the current nomenclature. We have replaced “*Streptococcus bovis*” with “*Streptococcus gallolyticus*” in Table 2 (p. 16). We have also reviewed the entire manuscript to ensure that the updated name is used consistently and correctly italicized throughout. We appreciate your careful review and trust that this correction meets your expectations.
